# Prevalence and predictors of institutional delivery among pregnant mothers in Biharamulo district, Tanzania: a cross-sectional study

**DOI:** 10.11604/pamj.2015.21.51.6347

**Published:** 2015-05-25

**Authors:** Kihulya Mageda, Elia John Mmbaga

**Affiliations:** 1 Biharamulo District Council, Department of Health, Biharamulo, Kagera, Tanzania; 2 Department of Epidemiology and Biostatistics, Muhimbili University of Health and Allied Sciences, Dar Es Salaam, Tanzania

**Keywords:** Institution delivery, home delivery, antenatal care services

## Abstract

**Introduction:**

Giving birth in a health facility is associated with lower maternal mortality than giving birth at home. A recent Tanzania Demographic Health survey showed that, although more than 90% of pregnant women attended at least one antenatal clinic visit, only 50% of pregnant women delivered at a health facility. The aim of this study was to document the magnitude and predictors of institutional delivery in order to assist in setting priorities and developing appropriate intervention measures to reduce maternal mortality.

**Methods:**

We conducted a cross-sectional study of women in Biharamulo district who delivered during the year preceding the survey. Multistage sampling was used to obtain 598 participants. A structured questionnaire was used to collect data. Bivariate and multivariate analysis was performed.

**Results:**

56% of women delivered in a health facility. Factors most strongly associated with institutional delivery were past care experience (aOR=265.1, 95%CI 28.6-2466.7), advice from health care provider to deliver at a health care facility (aOR=29.2, 95%CI 2.9-291.5), decision making on health care seeking on a pregnancy (aOR=7.1, 95%CI 2.7-19.0), maternal education (aOR=6.7, 95%CI 2.3-20.0), first antenatal care visit at <16 weeks (aOR=2.4, 95%CI 1.0-5.1), stable maternal income (aOR=2.3, 95%CI (1.1-4.7), and distance to facility <5 km (aOR 2.3 (95%CI 1.3-3.9).

**Conclusion:**

The prevalence of institutional delivery in Biharamulo District remains low. To raise the prevalence, the district should implement measures to make institutional delivery the preferred option for pregnant women. These measures should include encouraging women to make early antenatal care visits and make plans with their spouses for institutional delivery, reducing costs, improving the experience for women undergoing delivering in a healthcare facility, and consider locating new facilities closer to the women who need them.

## Introduction

Maternal mortality rate is a key indicator of health status of a population. In 2013, the World Health Organization defined maternal mortality as the death of a woman while pregnant or within 42 days of termination of pregnancy, irrespective of the duration and site of pregnancy (uterine or extra uterine) from any cause related to or made worse by the pregnancy or its management [1]. Achieving good maternal health requires quality reproductive health services and a series of well-timed interventions to ensure a women's safe passage to motherhood. Failure to provide these services results in hundreds of thousands of needless deaths each year. Every year, nearly half a million women and girls needlessly die as a result of complications during pregnancy or childbirth, and 99% occur in developing countries [2]. In developing countries many women do not have access to skilled personnel during child birth. This lack of skilled attendance is another factor responsible for high maternal and infant mortality [3]. Health facilities can provide proper medical attention and hygienic conditions during delivery and can reduce the risk of complication and infection [4]. Health facility delivery among pregnant mothers can be increased through antenatal clinic (ANC) attendance by providing reproductive health education and services.

A recent report from the Tanzania Ministry of Health and Social Welfare established that although more than 90% of pregnant women attended at least one antenatal clinic visit, only 62% of pregnant woman delivered at a health facility [5]. In Biharamulo district, only 46% of pregnant women delivered at the health facility. Many individual, health facility, and community factors have been suggested to explain the lower institutional delivery observed. However, little has been done to identify important population-specific factors contributing to the observed lower institutional delivery in the district. Identifying these factors is key to developing population-specific intervention strategies to improve institutional delivery and consequently reduce child and maternal morbidity and mortality. Although the most recent TDHS showed a decline in the maternal mortality ratio from 578 to 556 deaths per 100,000 live births [6], it is still unacceptably high in Tanzania. Lack of health facility delivery contributes to the maternal mortality because most births that occur at home are not assisted by skilled attendants. Knowing the magnitude and predictors of institutional delivery is necessary to assist in setting priorities and developing appropriate intervention measures to reduce maternal mortality. We therefore aimed to provide such information by collecting and analyzing data from a cross-section of women who delivered a child during the year preceding the survey in Biharamulo district of Kagera region, Tanzani

## Methods

### Study design and population

We conducted a cross-sectional study of women who delivered in the past one year preceding the survey in Biharamulo district.

### Study area

Biharamulo District is one of the 8 districts of Kagera Region, and has an area of 5,627 square kilometers and a total population of 205,500 people. The district has 2 divisions, 15 wards, 74 villages, 384 hamlets, and 25 health facilities (1 hospital, 4 health centers, and 20 dispensaries). The number of women of reproductive age in the district was estimated to be 46,000; the pregnancy rate was 4% in a year with contraceptive use coverage of 15% in 2012 (Regional Annual Reproductive and Child Health Report).

### Sampling and data collection

Using the formula Z^2^p(100-p)/e^2^, the marginal error (e) of 5%, z at 95% confidence interval of 1.96, and prevalence (p) of institution delivery of 46%, we calculated the required sample size as 382; then we multiplied by 1.5 to account for design effect, non-response, and rounded up to a target enrollment of 600 women. Three wards were selected randomly from each division. From the six wards, a total of 15 villages were selected randomly, providing 36 hamlets. We attempted to enroll and interview all women from these 36 hamlets who delivered a child in the past year preceding the survey. We identified them by history of delivery in the past year and looking into their antenatal cards and child's age. From these women we collected information regarding their socio-economic characteristics such as age, education, marital status, occupation, residence, distance from health facility knowledge on the importance and institutional delivery, reproductive health (parity, number of live children, number of dead children, history about last pregnancy, ANC attendance during last pregnancy and the place of delivery for the last pregnancy. Maternal source of income was categorized as “regular” for those mothers with stable income such as employment or doing business, and “irregular” for those whose source of income depended on agriculture activities. We also collected information regarding mother's views of services provided at the health facility during the previous pregnancy and developed a variable of past care experience. This was categorized as “good” if the mother received free care during delivery, “satisfactory” if she contributed gloves/medicine due to facility out of stock and “bad” if she made full payment of a service. We developed a variable called “attitude” based on the mothers’ positive or negative responses to a series of questions on feelings toward institutional delivery. Moreover we developed a variable based on whether the mother received advice to deliver at health care facility or not. The “plan a place for delivery”variable captured whether the woman had given thought to and/or made a specific decision about where to deliver. The variable “decision maker” indicated whether the pregnant woman, her spouse, both, or someone else made the decision about delivery site. Twenty data collectors recruited from health personnel and performed the data collection.

### Ethical consideration

Ethical approval for this study was obtained from the Muhimbili University of Health and Allied Sciences (MUHAS) ethical committee. Participants offered their consent before participating in this study, and no name was recorded during data collection.

### Data analysis

Data were entered and analyzed using Stata software. Descriptive characteristics were used to describe the baseline characteristics of the mother. Categorical variables were cross tabulated to obtain proportions and prevalence ratios to look at the differences between proportions. Continuous variables were summarized with means and medians, and t-tests were used to test the differences. Variables such as maternal and paternal education/source of income, distance from clinic, attitude, parity, number of deliveries, age at first visit, number of visit, kind of advice, plan a place of delivery, decision making and past care experience were used to obtain adjusted odds ratios (aOR's) by logistic regression.

## Results

A total of 598 women were identified in the selected hamlets who had delivered during the year preceding the survey, and all were interviewed. The mean age was 28 years ±7.2 SD, ninety four percent were married/ cohabiting, fifty three percent had primary education or above, ninety seven percent were peasants, and fifty percent resided near a health facility. Among fathers, sixty eight percent had primary education or above. Among the respondents, ninety eight percent accessed ANC services at some time during their pregnancy, but only eleven percent made their first visit during the first 16 weeks of pregnancy, and only forty percent made four or more antenatal visits (Table 1, Table 2).

**Table 1:** Socio-demographic characteristics of the study participants, Biharamulo District, Tanzania 2012

Socio-demographic character	Number	Proportion (%)	Socio-demographic character	Number	Proportion (%)
Age (yrs)			Distance from nearest h/facility		
**Mean Age**	27.6±7.2SD		<15km	296	49.50%
15-19	65	10.87%	5-10km	153	25.59%
20-24	175	29.26%	10km & above	149	24.92%
25-29	131	21.91%	Time to walk to the nearest h/facility		
30-34	98	16.39%	0-29minutes	24	4.01%
35-39	83	13.88%	30-59minutes	131	21.91%
40+	46	7.69%	60-119minutes	165	27.59%
**Marital status**			129+minutes	278	46.49%
Married	563	94.15%			
Umarried	35	5.85%			
Maternal education					
Secondary	32	5.35%			
Primary	285	47.66%			
None	281	46.99%			
**Maternal source of income**					
Regular	89	14.88%			
Irregular	509	85.12%			
Paternal education					
Secondary&above	29	4.85%			
Primary	380	63.55%			
None	189	31.61%			
**Peternal source of income**					
Regular	50	8.36%			
Irregular	548	91.64%			
Presence of health facility					
Present	331	55.35%			
Absent	267	44.65%			

**Table 2:** Proportion of Mothers and Husband's character and Prevalence Ratio(PR) according to Institutional delivery among mothers who delivered a child in 2012

Variable	Institution Delivery	Home Delivery	Total	%Institution Delivery	Prevalence Ratio[95% conf.Interval]
***Age(years)***					
15-19	35	30	65	54%	1.0[0.7-1.3]
20-24	105	70	175	60%	1.1[0.8-1.4]
25-29	73	58	131	56%	1.0[0.7-1.3]
30-34	58	40	98	59%	1.1[0.8-1.4]
35-39	36	47	83	43%	0.8[0.5-1.1]
40+	26	20	46	57%	1
***Marital status***					
Married	315	248	563	56%	1.1[0.8-1.5]
Unmarried	18	17	35	51%	1
***Maternal education***					
Secondary	28	4	32	88%	1.8[1.5-2.1]
Primary	167	118	285	59%	1.2[1.0-1.4]
None	138	143	281	49%	1
***Paternal education***					
Secondary	24	5	29	83%	1.7[1.4-2.1]
Primary	217	163	380	57%	1.2[1.0-1.4]
None	92	97	189	49%	1
***Maternal source of income***					
Regular	71	18	89	80%	1.6[1.4-1.8]
Irregular	262	247	509	52%	1
***Paternal source of income***					
Regular	44	7	51	86%	1.6[1.4-1.9]
Irregular	290	258	548	53%	1
***Distance***					
<5km	205	91	296	69%	1.7[1.4-2.0]
5-10km	66	87	153	43%	1.0[0.8-1.4]
>=10km	62	87	149	42%	1
Attitude					
Positive	324	241	565	57%	2.1[1.2-3.7]
Negative	9	24	33	27%	1

### Prevalence of institution delivery

Among the 598 women interviewed, 333 (56%) delivered in a healthcare institution. Institutional delivery was frequent among mothers and fathers with secondary or more education (88% and 83%, respectively) and with regular sources of income (80% and 86%, respectively), but varied little by maternal age or marital status Women residing less than 5 km from a health facility were more likely to have an institution delivery than those who lived ≥5 km away (69% versus 42%). Women with a positive attitude about institutional delivery were more likely to deliver there than those with a negative attitude (57% versus 27%). Institution delivery was more common among women whose first antenatal visit was before 16 weeks gestation (75%), and among those who made more than four antenatal visits (65%). Although most women had planned a place for delivery, those who had not were much less likely to have an institutional delivery (18% vs. 58%). Women who jointly decided with their spouses on a place for delivery were more likely to have an institutional delivery than when the man alone, woman alone, or a relative made the decision (Table 2, Table 3, Table 4).

**Table 3:** Proportion of Mothers Past obstetric history and Prevalence Ratio(PR) according to Institutional delivery among mothers who delivered a child in 2012

Variable	Institution Delivery	Home Delivery	Total	%Institution Delivery	Prevalence Ratio[95% conf.Interval]
***Parity***					
1-2	110	59	169	65%	1.6[1.2-2.1]
3-4	89	78	167	53%	1.3[1.0-1.8]
5-6	67	52	119	56%	1.4[1.0-2.0]
7-8	40	35	75	53%	1.3[1.0-2.0]
9+	27	40	67	40%	1
***Number of deliveries***					
<2	114	63	177	64%	1.7[1.2-2.5]
3-4	90	79	169	53%	1.4[1.0-2.0]
5-4	63	52	115	55%	1.5[1.0-2.1]
7-8	36	45	81	56%	1.2[0.8-1.8]
9+	21	35	56	37%	1
***Number of surviving children***					
<2	118	66	184	64%	1.8[0.8-3.9]
2-3	60	43	103	58%	1.6[0.7-3.6]
4-5	82	77	159	52%	1.4[0.6-3.1]
6-7	47	44	91	52%	1.4[0.6-3.1]
8-9	22	28	50	40%	1.2[0.5-2.8]
10+	4	7	11	36%	1
***Number of died chidren***					
<2	318	256	574	55%	0.7[0.4-1.3]
2-3	5	7	12	42%	0.6[0.2-1.3]
4-5	7	1	8	87%	1.2[0.6-2.2]
6+	3	1	4	75%	1
***Access RCH services***					
yes	329	260	589	56%	1.3[0.6-2.6]
no	4	5	9	445%	1
***Age at first visit (weeks)***					
<16	49	16	65	75%	1.6[1.2-2.1]
16-23	166	120	286	58%	1.2[1.0-1.6]
24-31	80	87	167	48%	1.0[0.8-1.3]
32+	38	42	80	48%	1
***Number of visits***					
once	9	10	19	47%	1
twice	45	56	101	45%	0.9[0.6-1.6]
thrice	126	100	226	56%	1.2[0.7-1.9]
four times	117	77	194	60%	1.3[0.8-2.1]
above four times	31	16	47	65%	2.1[1.2-3.8]

**Table 4:** Proportion of Mothers Past obstetric history and Prevalence Ratio(PR) according to Institutional delivery among mothers who delivered a child in 2012

Variable	Institution Delivery	Home Delivery	Total	%Institution Delivery	Prevalence Ratio[95% conf.Interval]
***Advice given***					
yes	315	246	561	56%	1.2[0.8-1.6]
no	14	17	31	45%	1
Missing	4	2	6		
***Kind of advice***					
Deliver at h/facility	318	241	559	57%	4.3[1.2-15.5]
Deliver at home	2	13	15	8%	1
Missing	13	11	24	54%	
***Plan Place for delivery***					
yes	327	237	564	58%	3.3[1.6-6.8]
no	6	28	34	18%	1
***Decision Making***					
Both	54	15	69	78%	1.9[1.5-2.2]
Husband	120	55	175	69%	1.6[1.4-1.9]
relative	39	30	69	57%	1.3[1.0-1.7]
Myself	120	164	284	42%	1
***Any medical experience***					
Good	215	98	313	69%	57.7[8.2-405.5]
Satisfactory	117	84	201	58%	48.9[6.9-344.3]
Bad	1	83	84	1%	1

### Factors associated with institution delivery

Factors significantly associated with institutional delivery on bivariate model analysis included any past medical care, kind of advice, plan a place for delivery, number of visits, attitude, decision making, maternal education, number of deliveries, paternal education, distance, paternal source of income, parity, age at first visit and maternal source of income (Table 2, Table 3, Table 4). In the multivariate logistic regression analysis the variables found to be independently associated with instutional delivery were past care experience, kind of advice, decision making, maternal education, age at first visit and distance, (Table 5, Table 6). Mothers who previously received good and satisfactory delivery service care were more likely to have institution delivery, respectively. Those who were advised by a health care worker to deliver at health care services were more likely to have institutional delivery. Women who shared in the decision about delivery location with their husbands were most likely to have institutional delivery. Mothers with secondary education were more likely to have an institutional delivery. Mothers with regular sources of income were more likely to deliver at a health care facility than those with irregular sources of income. Mothers residing within 5 km of a health care facility were twice more likely to have an institutional delivery than those living further away. Pregnant mothers who made their first antennal visits at less than 16 weeks gestation were more likely to have institutional delivery.

**Table 5:** Multivariate logistic regression analysis of variable associated with Institutional delivery utilization services in Biharamulo district, Tanzania, 2012

Variable	Institution Delivery	Home Delivery	cOR[95% conf.Interval]	aOR[95% conf.Interval]	P-value
**Maternal education**					
Secondary	28	4	7.0[2.4-20.4]	6.7[2.3-20.0]	0.001
Primary	167	118	1.5[1.1-2.1]	1.5[0.1-1.2]	0.015
None	138	143	1	1	
**Paternal education**					
Secondary	24	5	1.5[1.0-2.1]	1.8[0.5-7.2]	0.397
Primary	217	163	5.0[1.8-13.6]	1.0[0.7-1.6]	0.893
None	92	97	1	1	
**Maternal source of income**					
Regular	71	18	3.7[2.1-6.4]	2.3[1.1-4.7]	0.027
Irregular	262	247	1	1	
**Paternal source of income**					
Regular	44	7	5.3[2.3-12.0]	2.8[0.9-9.4]	0.088
Irregular	290	258	1	1	
Distance					
<5km	205	91	3.2[2.1-4.8]	2.3[1.3-3.9]	0.003
5-10km	66	87	1.1[0.7-1.7]	1.0[0.6-1.9]	0.923
>=10km	62	87	1	1	
Attitude					
Positive	324	241	2.8[1.3-6.3]	0.9[0.3-2.9]	0.806
Negative	9	24	1	1	
Parity					
1-2	110	59	2.8[1.5-5.0]	0.8[0.1-4.8]	0.792
3-4	89	78	1.7[0.9-3.0]	0.7[0.1-8.7]	0.768
5-6	67	52	1.9[1.0-3.5]	0.6[0.0-11.7	0.757
7-8	40	35	1.8[0.9-3.6]	2.0[0.1-6.7]	0.688
9+	27	40	1	1	
**Number of delivery**					
1-2	114	63	3.0[1.6-5.6]	1.0[0.2-6.1]	0.999
3-4	90	79	1.8[1.0-3.5]	1.1[0.1-14.4]	0.929
5-6	63	52	2.0[1.0-3.9]	1.0[0.6-18.5]	0.998
7-8	36	45	2.2[1.1-4.5]	0.1[0.0-4.5]	0.264
9+	21	35	1	1	
**Age at first visit(weeks)**					
<16	49	16	3.2[1.5-6.6]	2.5[1.1-5.1]	0.032
16-23	166	120	1.5[0.9-2.5]	0.6[0.3-1.3]	0.214
24-31	80	87	1.0[0.6-1.7]	1.0[0.6-1.7]	0.993
32+	38	42	1	1	

**Table 6:** Multivariate logistic regression analysis of variable associated with Institutional delivery utilization services in Biharamulo district, Tanzania, 2012

Variable	Institution Delivery	Home Delivery	cOR[95% conf.Interval]	aOR[95% conf.Interval]	P-value
**Number of visit**					
Once	9	10	1	1	
Twice	45	56	0.9[0.3-2.4]	0.6[0.2-2.4]	0.467
Thrice	126	100	1.4[0.6-3.6]	0.8[0.2-3.1]	0.729
Four times	117	77	1.7[0.7-4.4]	0.9[0.2-3.4]	0.844
More than four	31	16	2.2[0.7-6.4]	0.7[0.2-3.4]	0.679
**Kind of advice**					
Deliver at h/facility	318	241	15.8[2.0-122.0]	29.22[2.9-291.5]	0.001
Deliver at home	2	13	1	1	
**Plan Place for delivery**					
Yes	327	237	3.1[1.4-7.0]	1.7[0.5-5.5]	0.404
No	6	28	1	1	
**Decision Making**					
Both	54	15	4.5[2.4-8.5]	7.1[2.7-19.0]	0.001
Husband	120	55	2.9[1.9-4.3]	1.6[1.0-2.5]	0.007
Relative	39	30	1.7[1.0-3.0]	1.0[0.5-2.0]	0.957
Myself	120	164	1	1	
**Any medical experience**					
Good	215	98	171.3[23.5-124.7]	265.4[28.6-2466.5]	0.001
Satisfactory	117	84	111.8[15.2-821.2]	187.9[20.2-1744.5]	0.001
Bad	1	83	1	1	

## Discussion

In developing countries, although more pregnant woman axre attending ANCs over time, skilled delivery services remain underutilized [7]. In other studies, the predominant factors associated with not utilizing delivery services reported from several studies include lack of education of the mother, financial limitation and rural residence, tradition and culture, poor quality delivery at the health facility, and delay in starting antenatal care follow-up [2, 4, 8–10]. In this study, we found that 56% of women delivered in a health facility. This prevalence is higher than the prevalence in studies conducted in Ethiopia, Uganda and India [11–14]. However it is still relatively low compared to the national average (62%) in Tanzania survey of 2012. Thus, additional efforts are needed to ensure that the Tanzanian national target of 80% is achieved [5]. Women who had bad any medical care experience stopped using institutional delivery services in this study. This finding is consistent with a study done in Thailand that good care influenced women to deliver at a health care facility [15]. In Tanzania, quality of care can be influenced by the District Medical Officer and the facility staff by monitoring and evaluating antenatal care services, and ensuring that pregnant women receive high quality services. Improving the quality of care in health facilities, making sure that all essential medical supplies and medicine are in place and handling women's pregnancies properly should improve the reputation of institutional delivery and ultimately increase its utilization. Advice from healthcare workers during antenatal care increases a woman's use of institutional delivery. In Tanzania, healthcare workers can do more to provide good information regarding safe health care delivery and encourage women to deliver at health facility. A study done in Ethiopia found that proper counseling and advice to deliver at health care facility increased institutional delivery [16]. Another factor increases institution delivery in the current study is whether decision for a place of delivery is made by both parents. This finding is similar to the studies done in Ethiopia and Bangladesh [17, 18]. In most African communities, the male usually holds the economic power of the family. If men are encouraged to participate in antenatal care services and other reproductive health services, they may influence women to utilize institution delivery and may assist in financing other costs. Maternal education was significantly associated with institution delivery in this study. Education helps partners to understand well the danger signs and complications during pregnancy and delivery. Furthermore it helps to overcome some of taboos and other traditional customs that hinders utilization of health facilities. The finding that education was a significant factor influencing institution delivery is consistent with studies done in Nepal [19], Ethiopia [11, 20], and Kenya [21, 22]. Yalem documented that education is a key factor for improving maternal health care and access. He found that having five or more years of education was a predictor for both ANC use and institutional delivery [21].

Stable maternal source of income was significantly associated with institutional delivery in this study. In principle, health care in Tanzania for pregnant women is free. However, healthcare facilities occasionally run out of stock, requiring a pregnant woman to incur some expenses ranging from 10 to 15 USD for supplies or services. In addition, some women need to pay transportation fare to travel from home to the health facility. Thus, pregnant women with good sources of income are better able to pay for transport and unexpected healthcare facility costs. A study done in Ethiopia also found that women with stable income were more likely to utilize institutional delivery [23]. The current study also revealed that the mother's distance from home to the nearest health care services was associated with institutional delivery. Mothers who resided far from the health care facility were more likely to choose to deliver at home-as distance decreases, institutional delivery increases. Shorter distance may be related to less time taken by the mother to reach the facility when labour starts and lower transportation cost. But here the district medical officer and the respective clinical facility in charge has less control over distance. This current finding was similar to studies done in India [4], Laos [8], Ethiopia [12], Nepal [24], and Bangladesh [18, 25] that the mother's distance from the nearest health care facility predicts the choice of place of delivery. However, a study done in Kenya [25] suggested that physical accessibility rather than distance was associated with place of delivery. This study also revealed that the time of first ANC visit is associated with institution delivery. Women who started visiting an ANC earlier were more likely to deliver at a healthcare facility than women who started later. Women who start earlier ANC services are more likely to receive adequate prenatal care [14]. In addition, they are more likely to receive health education messages about pregnancy and safe delivery. They may develop a positive attitude about delivery at an institution. This finding is similar to the study done in Ethiopia that time of first ANC visits was associated with institution delivery [7]. Pregnant women should be encouraged for use institutional delivery during their antenatal visits [26]. Two strengths of this study are the large sample size and 100% response rate, thus reducing random error and potential for selection bias. A potential strength and limitation of the study was that the interviewers were all health workers who conducted the interviews at the respondent'home. On the one hand, the interviewers were able to review the respondents’ antenatal cards. This is the cross-section study design it is difficult to determine if any of the variables influence, determine or modify the outcome. Granted the exposure determined retrospectively does occurred before outcome but still I find the interpretation more complicated.

## Conclusion

The prevalence of institutional delivery in Biharamulo District is still well below the National target of 80% health care facility delivery. The factors associated with institution delivery in the District were past care experience, kind of advice, decision making, maternal education, and maternal source of income, distance, and timing of first ANC visit. The district should develop and implement strategies to improve the care and experience and reduce the costs of women attending health care facilities. Health workers should encourage women to make early visits at ANC and planning a place for delivery. Both partners should be encouraged to attend antenatal visits together and joint decision making. The Local Government should increase efforts to ensure health facility/health workers availability close to the people who need it.
